# What’s in a voice? Dolphins do not use voice cues for individual recognition

**DOI:** 10.1007/s10071-017-1123-5

**Published:** 2017-08-08

**Authors:** Laela S. Sayigh, Randall S. Wells, Vincent M. Janik

**Affiliations:** 1 School of Cognitive Science, Hampshire College, Amherst, MA 01002 USA; 2 Biology Department, Woods Hole Oceanographic Institution, Woods Hole, MA USA; 3 Chicago Zoological Society’s Sarasota Dolphin Research Program, c/o Mote Marine Laboratory, Sarasota, FL USA; 4 Scottish Oceans Institute, School of Biology, University of St. Andrews, St. Andrews, UK

**Keywords:** Dolphin, Playback experiment, Non-signature whistle, Voice cues, Individual recognition

## Abstract

Most mammals can accomplish acoustic recognition of other individuals by means of “voice cues,” whereby characteristics of the vocal tract render vocalizations of an individual uniquely identifiable. However, sound production in dolphins takes place in gas-filled nasal sacs that are affected by pressure changes, potentially resulting in a lack of reliable voice cues. It is well known that bottlenose dolphins learn to produce individually distinctive signature whistles for individual recognition, but it is not known whether they may also use voice cues. To investigate this question, we played back non-signature whistles to wild dolphins during brief capture-release events in Sarasota Bay, Florida. We hypothesized that non-signature whistles, which have varied contours that can be shared among individuals, would be recognizable to dolphins only if they contained voice cues. Following established methodology used in two previous sets of playback experiments, we found that dolphins did not respond differentially to non-signature whistles of close relatives versus known unrelated individuals. In contrast, our previous studies showed that in an identical context, dolphins reacted strongly to hearing the signature whistle or even a synthetic version of the signature whistle of a close relative. Thus, we conclude that dolphins likely do not use voice cues to identify individuals. The low reliability of voice cues and the need for individual recognition were likely strong selective forces in the evolution of vocal learning in dolphins.

## Introduction

Most mammals, including humans, can accomplish recognition of other individuals by means of “byproduct distinctiveness” (Boughman and Moss 2003), whereby the shape and size of the vocal tract render vocalizations produced by a given individual uniquely identifiable (e.g., bats: Balcombe 1990; primates: Belin 2006; Rendall et al. 1996; Snowdon and Cleveland 1980; sheep: Searby and Jouventin 2003; fur seals: Charrier et al. 2002). Such identifying features have been referred to as “voice cues.” Sound production in odontocete cetaceans (toothed whales, dolphins, and porpoises) does not involve the larynx as in other non-human mammals, but instead involves the vibration of membranes in the “monkey/phonic lips-dorsal bursae” complex of the nasal region (Au et al. 2012; Cranford et al. 1996; Madsen et al. 2010, 2012; Ridgway and Carder 1988). These gas-filled nasal sacs are susceptible to pressure changes associated with changes in depth (Ridgway et al. 2001), which have been suggested to result in a lack of reliable voice cues in these animals (Tyack 2000). A lack of voice cues could have been a driving force in the evolution of individually distinctive signature whistles that are found in many delphinid species (e.g., common bottlenose dolphins, *Tursiops truncatus*, Caldwell et al. 1990; Indo-Pacific bottlenose dolphins, *Tursiops aduncus*, Gridley et al. 2014; common dolphins, *Delphinus delphis*, Caldwell and Caldwell 1968; Pacific white-sided dolphins, *Lagenorhynchus obliquidens*, Caldwell and Caldwell 1971; spotted dolphins, *Stenella plagiodon*, Caldwell et al. 1973; Pacific humpback dolphins, *Sousa chinensis*, van Parijs and Corkeron 2001; Guiana dolphins, *Sotalia guianensis*, de Figueiredo and Simão 2009; Lima and Le Pendu 2014). Such “designed individual signatures” (Boughman and Moss 2003) have not been documented in any non-human mammals other than delphinid cetaceans to date, and may share some of the functions of human names (Janik and Sayigh 2013).

Signature whistles were first described by Melba and David Caldwell in the 1960s (Caldwell and Caldwell 1965). In studies of bottlenose dolphins under human care, they found that each dolphin predominantly produced one unique whistle contour (pattern of frequency changes over time) when isolated from conspecifics. Although signature whistles are defined as the predominant whistle that occurs in isolation (Caldwell et al. 1990), they are also important vocalizations when dolphins are free-swimming. Cook et al. (2004) found that approximately 50% of whistles produced by undisturbed groups of dolphins in Sarasota Bay, Florida, were either signature or probable signature whistles. The Caldwells’ pioneering work has been upheld by numerous other researchers over the past 5 decades (e.g., Agafonov and Panova 2012; Bruck 2013; Burdin et al. 1975; Cook et al. 2004; Esch et al. 2009a, b; Fripp et al. 2005; Harley 2008; Janik et al. 1994, 2006, 2013; Janik and Slater 1998; King et al. 2013, 2014; Luís et al. 2015; Miksis et al. 2002; Nakahara and Miyazaki 2011; Papale et al. 2015; Quick and Janik 2012; Sayigh et al. 1990, 1995, 1999, 2007; Sidorova et al. 1990; Sidorova and Markov 1992; Tyack 1986; Watwood et al. 2004, 2005). Probably signature whistles will continue to be identified in additional species as techniques are utilized for identification of vocalizing individuals, such as hydrophone arrays (e.g., Quick et al. 2008) and acoustic tags (e.g., Johnson and Tyack 2003). Such identifications will also be facilitated by a recently described technique to identify signature whistles from single hydrophone recordings of multiple individuals, which uses the temporal patterning of signature whistles to differentiate them from non-signature whistles (Janik et al. 2013).

**Fig. 1:**
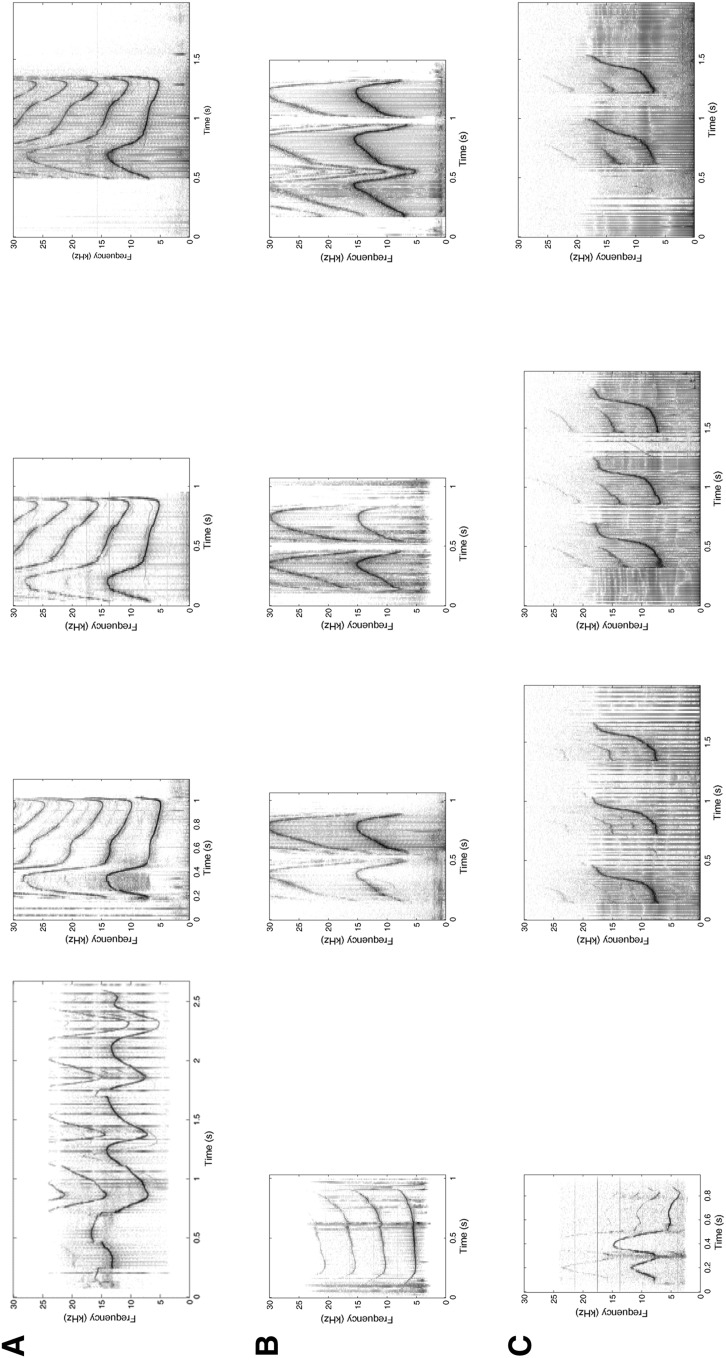

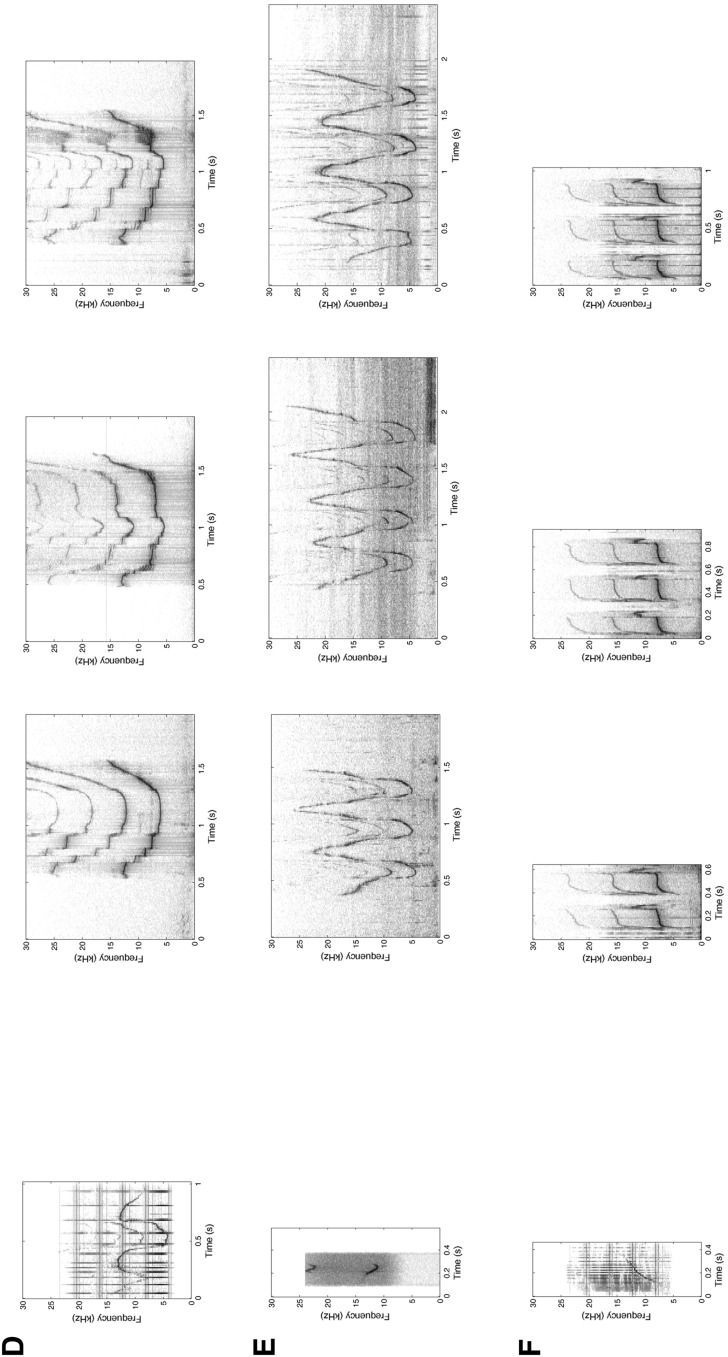
Spectrograms of non-signature and signature whistles produced during capture-release by six different dolphins (**a**–**f**). For each dolphin, the first exemplar is a non-signature whistle and the remaining three are randomly selected signature whistles. Frequency (up to 30,000 Hz) is on the *y* axes, and time in seconds is on the *x* axes. Spectrogram settings included a 1024-point Hanning window with 50% overlap

Non-signature whistles (which have also been called “variant” whistles) have been defined as any whistle other than the signature (Caldwell et al. 1990). Unlike signature whistles, non-signature whistles tend to be highly variable in contour, with many different contour types, some of which are shared among individuals (Janik and Slater 1998). To date, very little research has focused on non-signature whistles, and next to nothing is known about how they function in the natural communication system of dolphins. Sayigh et al. (1990) found that males in the long-term resident Sarasota Bay, Florida, bottlenose dolphin community (Wells 2014) tended to produce more non-signature whistles than did females, although this was based on a small sample of 12 dolphins. They speculated that females may be selected to produce more stereotyped vocalizations than males, as one of the primary roles of signature whistles appears to be maintenance of contact between a mother and her calf. Example spectrograms of non-signature whistles from six different dolphins are shown in Fig. 1. For comparison purposes, examples of signature whistles from the same six individuals are also shown.

Dolphins can recognize one another by means of their signature whistles (Sayigh et al. 1999) and can also recognize individuals by hearing the frequency contour of the signature whistle alone, with all potential voice cues removed (Janik et al. 2006). However, the possibility that dolphins may use voice cues as an additional means of recognizing individuals remains open. We hypothesized that non-signature whistles, given their lack of stereotypy and the fact that general types can be shared across individuals, would be recognizable to other dolphins only if they contained characteristic voice cues identifying the vocalizer. We tested this hypothesis by playing back non-signature whistles to wild dolphins during brief capture-release events in Sarasota Bay, Florida, using the identical protocol as in Sayigh et al. (1999) and Janik et al. (2006). These earlier studies found that target dolphins turned more toward the playback speaker in response to whistles from related versus unrelated (but familiar) individuals. Thus, we predicted that dolphins would turn more toward the speaker in response to non-signature whistles of related versus unrelated (but familiar) individuals if they were able to recognize non-signature whistles by means of voice cues.

## Methods

Our study was carried out with the resident community of bottlenose dolphins (*Tursiops truncatus*) near Sarasota Bay, Florida, USA. This dolphin community has been the focus of a long-term research program since 1970 (Wells et al. 1987; Scott et al. 1990). The community of about 160 resident dolphins spans up to five concurrent generations and includes individuals up to 67 years of age (Wells 2003, 2014; R. S. Wells, unpublished data). Since 1984, acoustic recordings of these dolphins have been made during occasional brief capture-release events, at which animals are assessed for various health and basic biological parameters (Wells and Scott 1990; Wells et al. 2004). During capture-release, a 500 × 4 m net is deployed from a small outboard vessel in water that is generally less than 2 m in depth. This creates a net corral that contains a small group (generally 1–4) of dolphins for short (1–4 h) periods of time. Throughout this time, while animals are either being held in the net corral or being examined out of the water, animals are recorded with suction cup hydrophones placed on the melon (forehead). This results in recordings that are generally high in signal-to-noise ratio. Whistles were recorded with hydrophones that were either custom built at the Woods Hole Oceanographic Institution or built by High Tech, Inc. (Gulfport, MI). Recordings were made onto a variety of different media over the years. From 1984 to 1989, Marantz or Sony stereo-cassette recorders were used (frequency response approximately 20–20,000 Hz), followed by Panasonic AG-6400 or AG-7400 videocassette recorders (frequency response approximately 20–32,000 Hz) through 2005. Since 2006, recordings have been made digitally, on a Sound Devices 744T recorder (sampled at 96 kHz). We now have a library of recordings of 272 dolphins, most of which have been recorded on multiple occasions (up to 18).

This recording library was used to select stimuli for the playback experiments. Typically dolphins produce large numbers of signature whistles during capture-release (e.g., Esch et al. 2009b), but non-signature whistles are occasionally produced as well. We selected a single non-signature whistle from each of 126 individual dolphins and used these to create 30-s playback sequences, with each containing 8–12 repetitions of the same non-signature whistle, depending on whistle length. The sequences thus contained approximately the same overall whistle content, as fewer exemplars were played of longer whistles. Overall stimulus durations (calculated by multiplying the number of stimuli presented by the length of each stimulus) were compared for related (mean = 6.3 s) versus unrelated stimuli (mean = 6.8 s) with a Wilcoxon signed-ranks test and were not found to be significantly different (*N* = 40, W = 362; *Z* = −0.38, *P* = 0.70).

A key aspect of this study is that we used the identical playback paradigm as in Sayigh et al. (1999) and Janik et al. (2006), so that results could be compared among the three sets of experiments. A target animal was presented with whistle stimuli from two familiar individuals, one related (as determined through long-term observations and confirmed through genetic testing) and one unrelated. Related individuals were usually mothers or independent offspring, but were occasionally siblings. The two stimulus animals had both associated with the target animal at similar levels over the previous two years, as calculated by coefficients of association (Cairns and Schwager 1987) derived from boat-based photographic identification survey data; these values were derived by dividing the number of sightings of two animals together by the total number of sightings of both individuals. When possible, the two stimulus animals were also matched for age and sex. Coefficients of association were compared for related (mean = 0.15) versus unrelated (mean = 0.05) pairings with a Wilcoxon signed-ranks test and were not found to be significantly different (*N* = 40, *W* = 220; *Z* = −1.56, *P* = 0.12).

As in the previous studies, the response variable measured was head turns toward the playback speaker. Playbacks were conducted during eight capture-release sessions from February 2004 through May 2014.

**Fig. 2:**
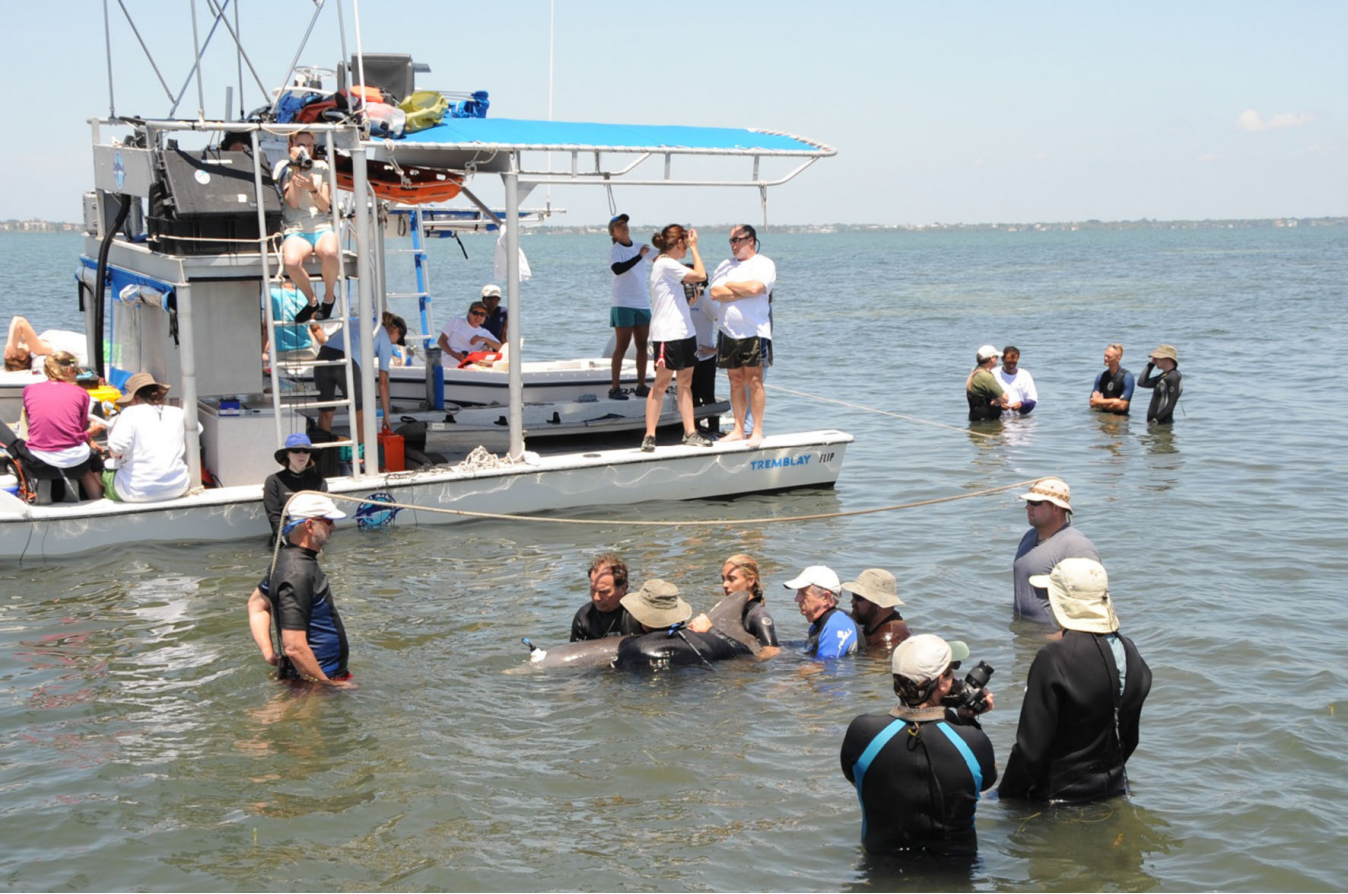
Playback experimental setup, showing the position of the videographer (sitting on *top of the ladder* on the boat), the playback speaker (held by the person at the *foot of the ladder* wearing a *blue hat*), and the target dolphin with a suction cup hydrophone on its melon. Photograph courtesy of Jim Schulz, Chicago Zoological Society, taken under National Marine Fisheries Service Scientific Research Permit No. 522-1785

All of the following field and analysis methods are identical to those of Sayigh et al. (1999) and Janik et al. (2006), but will be described briefly here. We used a LL9162 underwater speaker (Lubell Labs, Columbus, OH) connected to a car power amplifier to play back sounds to the dolphins. Sound files were played from a Dell laptop computer. The frequency response for the combined system was 240–20,000 Hz ±3 dB. The source level was pre-set to produce a received level at a 2-m distance from the speaker (the location of our experimental animal) that approximated the received level of whistles produced by a nearby dolphin (as judged by the experimenters). Individual stimuli in each playback were normalized for average amplitude. Playbacks were monitored with a hydrophone next to the speaker, and vocalizations of the target dolphin were recorded with a suction cup hydrophone attached to the melon for the duration of the experiment. If there were other animals present during a playback, either in the water or on the deck of the boat, their whistles were also recorded with suction cup hydrophones. Recordings were made with either a Panasonic AG-7400 video recorder (2004–2005) or a Sound Devices 744T digital recorder (2006–2014), with frequency responses described above. Playback sessions were recorded on either a Sony DCRTRN 320 or a Canon Vixia HFR40 digital video camera from a platform on a boat approximately 2 m above the water surface at the speaker position (Fig. 2). The speaker was suspended from an anchored boat at approximately 1 m depth, and approximately 2 m to one side of the target animal.

Dolphins were held loosely by about 4–5 handlers during the experiments but were able to turn their head freely in response to playbacks. All people holding the animal were blind to the playback sequence and could not hear in air when the stimuli were being played. Each target animal was held in position for a minimum of 5–10 min prior to an experiment so as to acclimate it to its surroundings. Each playback sequence lasted 30 s and was followed by 5 min of silence in order to document any continued responses by the target dolphin. We balanced the order of stimulus presentation, such that whistles from the related individual were played first in 20 trials and from the unrelated individual in 20 trials. We counted head turns greater than 20° toward the playback speaker within a 5.5-min period from the start of a playback as a response. Anything less than 20° was not counted because animals frequently moved back and forth within this range. Head turns were scored from video recordings of the playback sessions, without knowledge of the order of playback stimuli being presented. We compared the number of head turns toward non-signature whistles of related versus unrelated individuals. Each experiment was scored by at least two individuals, and scores were found to have a high level of agreement, with the overall statistical trends identical for both sets of scores (*W* scores from the Wilcoxon signed-ranks test comparing head turns to related versus unrelated stimuli were 290 and 292). Thus, again to be consistent with our earlier experiments, we used only one set of scores (those of author LS) for analyses. Whistle responses to playbacks were examined in Adobe Audition.

In addition to comparing responses to whistles of related and unrelated individuals, we examined effects of presentation order of the stimuli and sex of the target animal. As in our previous published studies (Sayigh et al. 1999; Janik et al. 2006), all data were tested with Wilcoxon signed-ranks tests. By keeping all aspects of this study identical to the previous studies, we were able to directly compare their results.

We also calculated effect size by dividing the Z score of the Wilcoxon signed-ranks test by the square root of the number of trials (Pallant 2007) and compared effect size in our published signature whistle playbacks to that observed in the current study. We compared the differences in number of head turns to related versus unrelated playbacks in our published studies to those observed in the current study with a Mann–Whitney *U* Test. We combined results from our natural (Sayigh et al. 1999) and synthetic (Janik et al. 2006) whistle experiments to obtain comparable sample sizes for these comparisons.

## Results

Dolphins did not respond differentially, in terms of head turns toward the speaker, to non-signature whistles of close relatives versus known unrelated individuals (*N* = 40, *W* = 292; *Z* = −0.64, *P* = 0.52; Table 1). The mean number of head turns toward non-signature whistles of related individuals was 10.8 versus 12.1 toward non-signature whistles of known unrelated individuals. These results contrast with our previously published signature whistle playback experiments (Sayigh et al. 1999; Janik et al. 2006), which showed significant differential responses to signature whistles of related versus known unrelated individuals (combined results: *N* = 34, *W* = 105; *Z* = −2.79, *P* = 0.005; mean values of 17.3 and 14.2 toward related and unrelated, respectively). Effect size in our combined signature whistle experiments was 0.33 (sample size of 34 paired trials), representative of a medium effect (Cohen 1988), whereas effect size in the non-signature whistle playbacks reported here (sample size of 40 paired trials) was only 0.07, representative of a negligible effect. In addition, the effect was in the opposite direction to that observed in our signature whistle playbacks; in the present study, animals turned more (although not significantly) to non-signature whistles of unrelated animals, whereas in our previous studies animals turned significantly more toward signature whistles of related individuals. The differences between the number of head turns to related versus unrelated playbacks in our previously published experiments (average difference = 3.1) and in the unrelated playbacks described in the current study (average difference = −1.3) were significantly different (*N*_1_ = 34, *N*_2_ = 40, *U* = 417.5; *Z* = −2.84, *P* = 0.004).

**Table 1:** Number of head turns (HT) toward the playback speaker following related and unrelated non-signature whistle stimuli, and coefficients of association (CoA) between target and stimulus animals; male target animals are in bold

Target animal ID	HT related stimulus	HT unrelated stimulus	CoA related stimulus	CoA unrelated stimulus
FB07	22	15	0	0.024
FB11	13	14	0.03	0.062
FB54	20	25	0.026	0.025
FB90	23	29	0.116	0.106
FB133^a^	13	10	0.149	0.142
FB135^a^	7	16	0.833	0.056
FB137^a^	18	14	0.885	0.106
FB137^a^	8	0	0	0.019
FB151	2	1	0.286	0.1
FB155	16	27	0.006	0.006
FB155^a^	19	15	0.039	0.07
FB159^a^	10	3	0.054	0.059
FB179	16	12	0.455	0.078
FB181	3	1	0.079	0.039
FB187^a^	13	13	0.174	0.153
FB199	5	6	0	0
FB203	4	6	0.078	0.073
FB205^a^	4	15	0.116	0.019
FB209	4	2	0.279	0.083
FB221^a^	1	0	0.327	0.078
FB229	1	5	0.4	0.034
FB241	3	0	0.19	0.132
**FB10**	16	16	0	0
**FB20**	1	0	0	0
**FB92** ^a^	5	8	0.033	0.025
**FB100** ^a^	3	3	0	0
**FB128**	3	1	0	0.031
**FB138**	19	27	0.072	0.113
**FB138** ^a^	5	6	0	0.018
**FB146**	28	35	0.008	0.024
**FB148**	5	8	0.041	0.069
**FB178**	18	18	0.045	0.073
**FB182**	7	10	0.032	0.011
**FB188**	45	46	0.056	0.059
**FB196**	20	51	0.019	0.01
**FB220**	8	5	0.024	0.075
**FB250**	6	9	0	0.027
**FB252** ^a^	5	4	0.094	0.037
**FB276**	9	6	0	0.014
**FB280** ^a^	3	1	0.986	0.136

^a^Experiments that were possibly compromised in some way (see text)

Presentation order had a marginally significant effect on head-turning behavior, with means of 12.5 head turns toward the first stimulus and 10.3 head turns toward the second stimulus (*N* = 40, *W* = 193.5; *Z* = −2.19, *P* = 0.028). However, this result was not significant after applying a Bonferroni correction to account for multiple testing of the same data. Sex of the target animal did not affect head-turning behavior. Females were found to show almost identical head-turning responses to non-signature whistles of related versus unrelated individuals (means of 9.9 and 10.6, respectively, *N* = 22, *W* = 111.5; *Z* = −0.139, *P* = 0.89). Males showed a slightly higher but still nonsignificant tendency to turn more toward non-signature whistles of unrelated animals (mean = 14.1) than to related animals (mean = 11.4; *N* = 18, *W* = 35; *Z* = −1.42, *P* = 0.16).

There were 14 experiments that may have been compromised by various factors (Table 1); thus data were also analyzed with these experiments excluded. Factors that may have compromised experiments included exposure to stimuli prior to the experiment (three cases), a playback stimulus that was from an animal in the same capture-release session (one case), a playback stimulus that contained crossover from another whistle (one case), and disruption of the playback setup during an experiment (one case). We also considered 7 experiments that were conducted after auditory evoked potential (AEP) experiments to be potentially compromised, given that these experiments involved playing back stimuli of varying frequencies through jawphones. A final factor is that three individuals (FB137, FB138, and FB155) each received two playbacks in two different years. By chance, two of the duplicate playbacks (FB137 and FB138) were among the possibly compromised playbacks mentioned above, so only one additional experiment was removed to account for these.

Results from the remaining 26 experiments were similar to those from the larger data set of 40 experiments. Dolphins did not respond differentially to non-signature whistles of close relatives (mean head turns 12.2) versus known unrelated individuals (mean head turns 14.4; *N* = 26, *W* = 103; *Z* = −1.34, *P* = 0.18; Table 1). Also similar to the overall data set, sex did not significantly affect head-turning responses. Females turned an average of 10.1 times in response to non-signature whistles of related animals and 11.0 times to whistles of unrelated animals (*N* = 13, *W* = 39.5; *Z* = −0.42, *P* = 0.67). Males again showed a slightly greater, although still nonsignificant, tendency to turn more toward non-signature whistles of unrelated animals (mean = 17.8) than to related animals (mean = 14.2; *N* = 13, *W* = 16.5; *Z* = −1.47, *P* = 0.14). Finally, presentation order still did not have a significant effect on head-turning responses when possibly compromised experiments were removed (mean head turns toward the first and second stimuli were 14.4 and 12.3; *N* = 26, *W* = 94.5; *Z* = −1.59, *P* = 0.11).

**Fig. 3:**
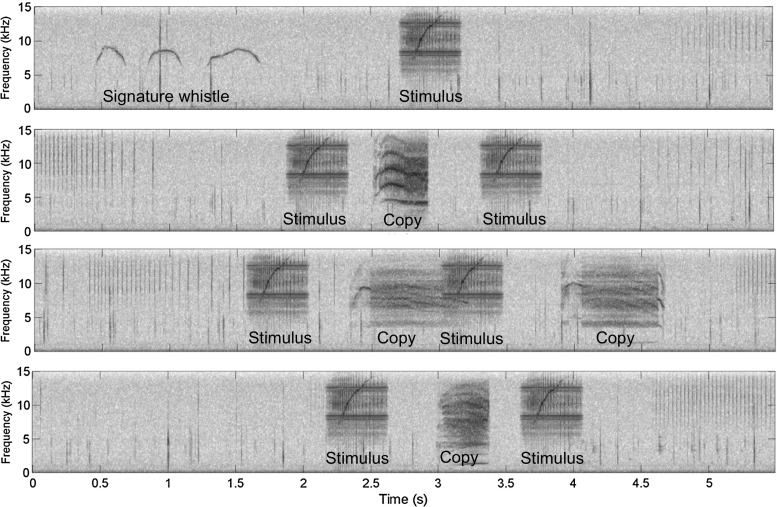
Spectrogram of copying of a noisy non-signature playback stimulus. A 22-s sequence is divided into four 5.5-s sections (*top panel* 0–5.5; *second panel* 5.5–11; *third panel* 11–16.5; *bottom panel* 16.5–22). The target animal’s signature whistle is visible at the beginning, followed by a stimulus presentation and then several stimulus-copy exchanges. Frequency (up to 15,000 Hz) is on the *y* axes, and time in seconds is on the *x* axes. Spectrogram settings included a 1024 point Hanning window with 50% overlap

**Fig. 4:**
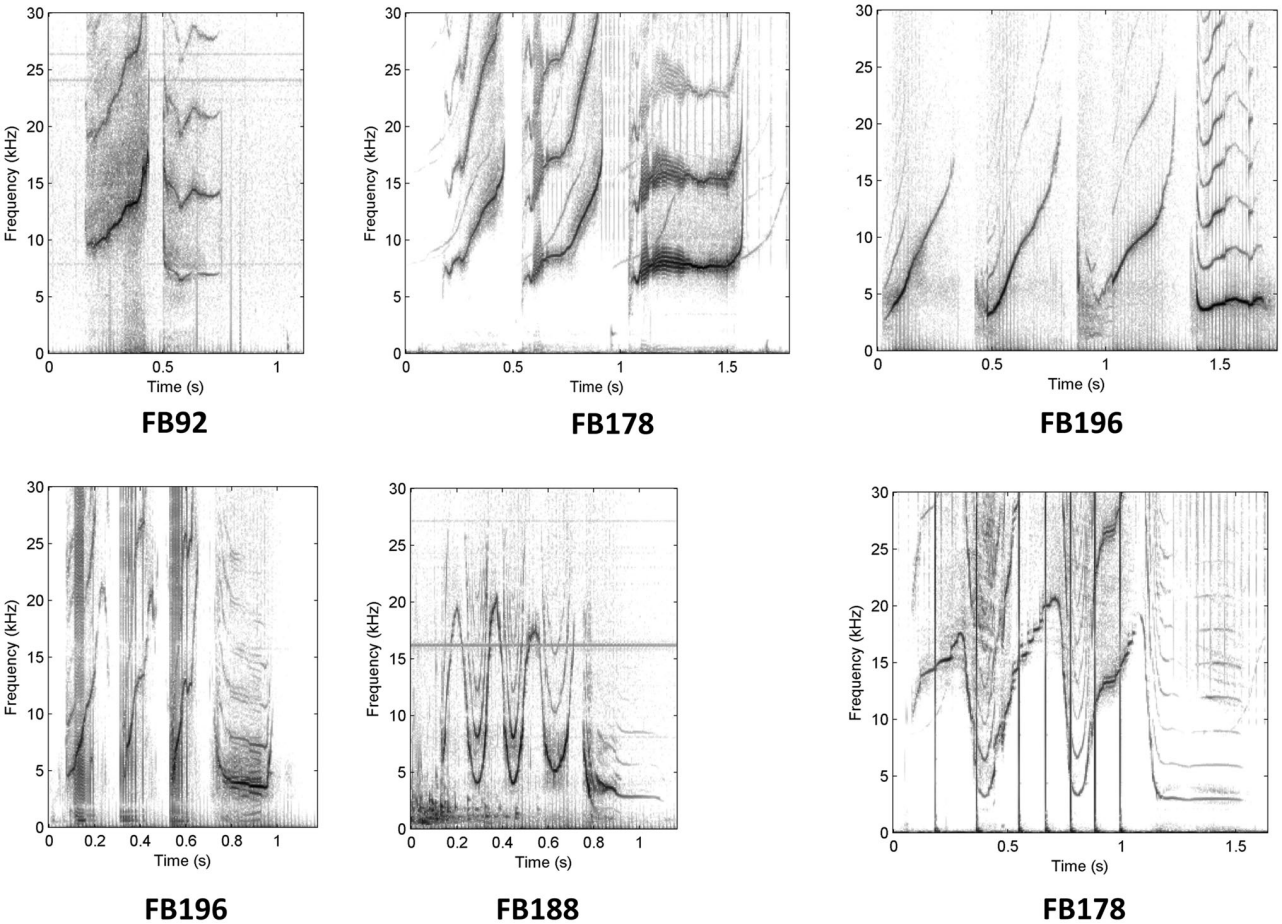
Spectrograms of similar non-signature whistle responses (called “M” whistles) to non-signature whistle playbacks by four different males (with two examples from each of two males, FB178 and FB196). Frequency (up to 30,000 Hz) is on the *y* axes, and time in seconds is on the *x* axes. Spectrogram settings included a 1024 point Hanning window with 50% overlap

Acoustic responses to non-signature whistle playbacks were also examined qualitatively. On 14 occasions, the target dolphin copied the playback stimulus (Fig. 3), as assessed by one experienced observer based on contour similarity. Six males were found to produce a similarly shaped non-signature whistle contour, which we called the “M” whistle based on its overall shape (Fig. 4). Although these whistle responses will be subjected to further quantitative analyses in the future, preliminary results suggest that target animals were equally likely to produce copies and “M” whistles in response to stimuli produced by related and unrelated animals (in fact, seven animals produced copies or “M” whistles in response to both stimuli), further suggesting that dolphins do not recognize voice cues.

## Discussion

Our results show that dolphins likely do not use voice cues to identify other individuals and instead carry out individual identification by means of the frequency modulation pattern of signature whistles alone. In experiments using protocols identical to the present study, dolphins turned significantly more toward the playback speaker when they heard playbacks of either natural (Sayigh et al. 1999) or synthetic (Janik et al. 2006) signature whistles of close relatives versus known unrelated animals, indicating that they recognized these whistles as identifying a specific individual. This difference was not observed in response to playbacks of non-signature whistles. Given that sound production is affected by water pressure and thus depth (Ridgway et al. 2001; Jensen et al. 2011), it is perhaps not surprising that voice cues would not be a reliable source of identity information in dolphins. But since dolphins live in fluid, fission–fusion societies (Wells et al. 1987; Connor et al. 2000) where vision is of limited usefulness due to poor water clarity and reduced light at depth, the need for reliable signals to identify one another is likely great. Signature whistles have been found to comprise approximately 50% of all whistles produced by free-ranging dolphins in Sarasota Bay, Florida (Cook et al. 2004), illustrating their importance in the natural communication system of dolphins.

Our results raise the question of how dolphins perceive copies of signature whistles, if voice cues are not involved in individual recognition. Several studies of whistle imitation have suggested that dolphins may include characteristic features in their copies that may render them recognizable as copies to other animals (e.g., Tyack 1986; King et al. 2013). In fact, the lack of recognizable voice cues may necessitate the use of such features in whistle copies in order for signature whistles to function as individual identifiers.

However, as is always the case with negative results, we cannot rule out alternative explanations for our data. One of the most obvious is the possibility that dolphins use non-signature whistles differently than signature whistles and thus are not motivated to respond in the same way to the two types of whistles. However, the capture-release situation is associated with increased stress for the animals (Esch et al. 2009b), and we think it is unlikely that animals would ignore the sudden and unexpected appearance of a close relative that could provide support, just because of the whistle type that it produced. Turning responses are reliable indicators of animals trying to explore a stimulus, and thus are what we would have expected in this context when we simulated the arrival of closely related individuals. As mentioned above, our previous studies showed that in the identical context, dolphins reacted strongly to the perceived presence of such allies when hearing their signature whistle (Sayigh et al. 1999) or even just a synthetic version of their signature whistle (Janik et al. 2006).

Another possible explanation for the observed lack of differential response is that we did not have a sufficient sample size to detect a significant effect. When we reached a sample size comparable to our earlier playback studies (Sayigh et al. 1999; Janik et al. 2006), we found nonsignificant results, but also found greater variability in the data than in our previous studies. We therefore decided to increase our statistical power by increasing the sample size. When sample size increased from 26 to 40 experiments, the results remained nonsignificant, and all trends in mean values of head turns stayed the same, giving us much more confidence in the robustness of the current results. Our current sample size (40) is greater than the combined sample (34) of our two earlier studies.

Dolphins showed a greater, although nonsignificant, tendency to respond to the first stimulus, suggesting a possible “surprise” effect. It is perhaps to be expected that animals might show initial interest to the unexpected appearance of another animal nearby. However, it is notable that in experiments involving signature whistles (Sayigh et al. 1999; Janik et al. 2006), responses were stronger to related animals regardless of presentation order. In other words, the greater salience of a whistle coming from a related individual masked any possible “surprise” effect. Further, in the present study, the mean coefficient of association between target and related animals used as stimuli was higher than between target and unrelated animals (although nonsignificant). This could have biased results in favor of stronger responses to related animals, yet this result still was not observed.

To exactly replicate the design of our earlier playback studies, we played back non-signature whistles in the same bout structure as we had played back signature whistle stimuli, with exemplars separated by 1 and 4 s of silence. Janik et al. (2013) found that while signature whistles typically occur in bouts with inter-whistle intervals of 1–10 s, non-signatures were typically separated by less than 1 or more than 10 s. We expect that if dolphins were able to recognize non-signature whistles of close relatives by means of voice cues, we would still see a differential response as we had in our signature whistle playback studies, regardless of bout structure. But the fact that we did not see a differential response, combined with the fact that our playback stimuli were presented with a bout structure typical of signature whistles, suggests that dolphins may have perceived these whistles as unfamiliar signature whistles. We are currently testing this hypothesis by conducting playback experiments with both non-signature and unknown signature whistles with bout structures typical of both signature and non-signature whistles.

The idea that dolphins perceived the playback stimuli as signature whistles of unfamiliar individuals also creates testable hypotheses about the unusual vocal responses to non-signature playbacks that we observed. We are examining whether copying of playback stimuli is more common in response to unfamiliar stimuli, and whether such copying may also have a social function. In addition, we are currently testing the hypothesis that “M” whistles may be produced by males in the presence of an unfamiliar animal, particularly in situations when the males are associating with a reproductively active female, which our preliminary data suggest to be the case.

Our findings suggest that non-signatures play a very different role in the dolphin communication system than do signature whistles: they do not convey individual identity, and there is potential that they convey some degree of context-specific information. While dolphins have been found to produce context-specific pulsed sounds (e.g., Connor and Smolker 1996; Janik 2000), there is yet no evidence for context-specific non-signature whistles. Given the impressive vocal learning skills of dolphins (Janik 2014), it is likely that non-signatures are also learned signals. Much remains to be discovered regarding how non-signature whistles function in the natural communication system of dolphins.

In summary, our results support the idea that dolphins differ from most other non-human mammals in their use of the frequency modulation pattern of individually distinctive signature whistles, rather than voice cues, for individual recognition. If voice cues became unreliable due to water pressure related changes in the shape of structures relevant for the quality of a sound, the need for individual recognition may have been a strong selective force in the evolution of vocal learning in dolphins (Janik 1999, 2009). Vocal production learning allows dolphins to produce novel whistles that are distinct and recognizable in the marine environment, where visibility is low, olfaction is less functional, and background noise is high. Thus, the combination of compromised voice cues and limitations on other sensory channels may have contributed to the relative prevalence of vocal production learning among marine versus (non-human) terrestrial mammals (Janik and Slater 2000).
